# Type 2 diabetes alters mesenchymal stem cell secretome composition and angiogenic properties

**DOI:** 10.1111/jcmm.12969

**Published:** 2016-09-19

**Authors:** Jonathan Ribot, Guavri Caliaperoumal, Joseph Paquet, Catherine Boisson‐vidal, Herve Petite, Fani Anagnostou

**Affiliations:** ^1^ Laboratory of Bioingénierie et Biomécanique Ostéo‐articulaires‐UMR CNRS 7052 Paris 7‐Denis Diderot UniversitySorbonne Paris CiteParisFrance; ^2^ INSERMUMR 1140Université Paris DescartesSorbonne Paris CiteParisFrance; ^3^ Department of PeriodontologyService of OdontologyPitié Salpêtrière Hospital et Hôtel‐Dieu Hospital AP‐HPU.F.R. of Odontology Paris 7‐Denis Diderot UniversitySorbonne Paris CiteParisFrance

**Keywords:** MSCs, diabetes type 2, secretome, angiogenesis, endothelial cells

## Abstract

This study aimed at characterizing the impact of type 2 diabetes mellitus (T2DM) on the bone marrow mesenchymal stem cell (BMMSC) secretome and angiogenic properties. BMMSCs from Zucker diabetic fatty rats (ZDF) (a T2DM model) and Zucker LEAN littermates (control) were cultured. The supernatant conditioned media (CM) from BMMSCs of diabetic and control rats were collected and analysed. Compared to results obtained using CM from LEAN‐BMMSCs, the bioactive content of ZDF‐BMMSC CM (i) differently affects endothelial cell (HUVEC) functions *in vitro* by inducing increased (3.5‐fold; *P* < 0.01) formation of tubule‐like structures and migration of these cells (3‐fold; *P* < 0.001), as well as promotes improved vascular formation *in vivo,* and (ii) contains different levels of angiogenic factors (e.g. IGF1) and mediators, such as OSTP, CATD, FMOD LTBP1 and LTBP2, which are involved in angiogenesis and/or extracellular matrix composition. Addition of neutralizing antibodies against IGF‐1, LTBP1 or LTBP2 in the CM of BMMSCs from diabetic rats decreased its stimulatory effect on HUVEC migration by approximately 60%, 40% or 40%, respectively. These results demonstrate that BMMSCs from T2DM rats have a unique secretome with distinct angiogenic properties and provide new insights into the role of BMMSCs in aberrant angiogenesis in the diabetic milieu.

## Introduction

Type 2 diabetes mellitus (T2DM) is reaching epidemic proportions worldwide. The persistent hyperglycaemic milieu in T2DM is associated with macro‐ and micro‐vascular complications affecting the heart, blood vessels, eyes, kidney and nerves, and the wound‐healing process in diabetic patients 1, 2, 3. Most of the T2DM pathological complications are associated with impaired vascularization and/or aberrant angiogenesis. Excessive and abnormal angiogenesis plays a pivotal role in diabetic retinopathy and nephropathy 4, 5, whereas deficient angiogenesis contributes to impaired wound healing and coronary collateral vessel development 1, 3, 6. Despite extensive research, the mechanism(s) behind the abnormal angiogenesis balance in diabetic patients is still poorly understood.

Scientific research has provided evidence that the dysfunction of different types of cells, including endothelial, peripheral blood and bone marrow mesenchymal stem cells (BMMSCs), is related to the reduced blood vessel regenerative potential in T2DM 7, 8, 9. Endothelial cells migrate in response to soluble mediators that regulate new blood vessel formation 10, but their function is impaired in the diabetic milieu 7, 11, 12. BMMSCs, whose phenotype is strongly determined by their specific environment, have the capacity to home in on sites of injury, proliferate and differentiate into multilineage cell types 13. The T2DM milieu affects the regenerative potential of BMMSCs as well as their proliferation 8, restricts their multipotency and impairs their capacity to augment post‐ischemic neovascularization in diabetic mice 14. BMMSCs exert a paracrine effect by the release of growth factors and cytokines, such as IGF‐1, which stimulate endothelial cell migration 15, inhibit endothelial apoptosis and promote angiogenesis 16. Moreover, by providing proangiogenic factors, BMMSCs create a favourable microenvironment that promotes neovascularization and tissue regeneration 13, 15, 17. Although important in this respect, the impact of T2DM on the BMMSC secretome and its angiogenic properties has not yet been characterized.

We hypothesized, therefore, that the BMMSC secretome in the diabetic milieu has a different composition and affects angiogenesis‐related endothelial cell (EC) functions, including proliferation and new blood vessel formation. We tested this hypothesis using Zucker diabetic fatty (ZDF) (Leprfa/fa) rats (a model of T2DM) and Zucker Lean control (LEAN) (Leprfa/+/Lepr+/+) rats (their age‐matched littermates) 18. By focusing on the impact of T2DM on BMMSC secretome functions, this study aimed at both elucidating the contribution of MSCs in diabetic‐related vascular complications and establishing relevant aspects for future cell therapies in diabetes.

## Materials and methods

### Animal models

Adult, 8‐week‐old, male, Swiss nude mice as well as adult, 12‐ to 13‐week‐old, male, obese Zucker fa/fa rats (ZDF) and their lean fa/+ littermates (LEAN) were purchased from Charles River (L'Arbresle, France). The animals were housed, operated and killed using procedures in accordance with the European Community Standards on the Care and Use of Laboratory Animals. The experimental procedures were approved by the Ethics Committee of the University Paris‐Diderot.

The diabetic condition of the rats used in this study was determined on the day of killing them. For this purpose, venous blood was collected, and the respective glycaemic state was evaluated using a glucometer (Roche Diagnostics, Meylan, France). Plasma concentrations of glucose and fructosamine were determined using commercially available kits (Roche Diagnostics) following the manufacturer's instructions.

### *In vitro* experiments

#### Isolation of rat mesenchymal stem cells (BMMSCs)

The femurs and tibiae from each rat were cleaned of connective tissues, and their respective epiphyses were removed to allow insertion of 23‐gauge needles connected to syringes containing serum‐free αMEM (Invitrogen, Cergy Pontoise, France). The cells present in the harvested marrow were then homogenized using complete medium composed of αMEM supplemented with 10% (v/v) foetal calf serum and 1% (v/v) antibiotic/antimycotic (ATB/ATM) solution (PAA Laboratories GmbH, Pasching, Austria). The isolated cells from rats were seeded at 5 × 10^5^ cells/cm² and cultured at 37°C in a humidified 5% CO_2_/95% air environment. After 2 days of culture, the supernatant media (containing non‐adherent cells) were discarded. Fibroblastic colonies (CFU‐F) appeared at day 5 of culture and were all pooled at day 12 (cell passage 1). For amplification of the LEAN‐ and ZDF‐BMMSC populations, the cells were seeded at 10 × 10^3^ cells/cm². The supernatant media were changed twice a week. BMMSCs at passage 2–3 were used for the experiments of this study.

#### Preparation of conditioned media

BMMSCs were seeded at 10^4^/cm² in a culture‐treated flask and cultured in calf serum‐free αMEM under standard cell culture conditions. After 24 hrs, the supernatant was collected, centrifuged (700 × g, for 4 min.), aliquoted and frozen at −80°C until further use. The concentration of total proteins in the conditioned medium (CM) of BMMSCs from both ZDF and LEAN rats was determined using the Bradford protein assay and following the manufacturer's instructions. For experiments with neutralizing antibodies, anti‐IGF‐1, anti‐LTBP1 or anti‐LTBP2 were added to CM (5 ng/mL) and maintained at 37°C for 1 hr before use in experiments with cells.

#### Culture of HUVECs

The effects of CM on endothelial cells were studied using human umbilical vein endothelial cells (HUVECs). HUVECs in EGM‐2 medium (Lonza, France) supplemented with 5% foetal calf serum and 1% (v/v) ATB/ATM were cultured in tissue‐culture flasks pre‐coated with 0.5% gelatin under standard cell culture conditions. When the cells reached 80% confluency, they were passaged by trypsinization (Trypsin/EDTA Solution, Life Technologies, Alfortville, Ile de France, France).

### Cell proliferation

Cells were seeded at the density of 3 × 10^3^ cells/cm^2^ in individual wells of 12‐well culture plates in αMEM containing 10% FBS. For each time‐point, BMMSCs were trypsinized, incubated with Trypan blue and counted using a Malassez chamber.

### Migration of human umbilical vein endothelial cells in Boyden chambers

HUVEC migration was determined using commercially available Boyden chambers (Corning Costar, Tewksbury MA, USA) whose two compartments were separated by polycarbonate membranes with 8 μm diameter pores. Aliquots of cells (50 × 10^3^ cells) in 100 μl of αMEM without foetal calf serum were placed in the upper chamber, and the various media of interest to this study were each placed in the bottom chamber. The cell migration experiments were conducted in a humidified, 37°C, 5% CO_2_/95% air environment for 24 hrs. The cells that had transversed but still adhered on the other side of the membrane separating the Boyden chamber were then stained *in situ* using May Grunwald‐Giemsa stain. All such membranes were excised, mounted on slides, visualized using light microscopy and photographed.

### Wound‐healing assay

HUVECs were seeded at a density that resulted in ~70–80% confluence on the bottom surface of individual wells of 12‐well tissue‐culture plates within 24 hrs of culture. At that time, each monolayer was scratched across the respective centre using a new 1‐ml pipette tip and was rinsed twice with phosphate‐buffered saline (PBS) to remove detached cells. The wounded HUVEC samples were then treated with CM from either BMMSCs of diabetic or control rats at 37°C for 6 hrs, rinsed twice with PBS and fixed using 4% paraformaldehyde for 30 min. The scar region on each cell monolayer was visualized using light microscopy and photographed before and after the 6‐hr interval. Comparison of the evidence on these two sets of micrographs was used to determine the migration of HUVECs. These data were expressed as the difference in the distance travelled by HUVECs from the edges of each scratch region towards the centre of the wounded area.

### Formation of tubular‐like structures by HUVECs on Matrigel

Each well of 48‐well tissue‐culture plates was coated with 10 mg/ml Matrigel (growth factor reduced Matrigel, VWR France) and maintained in a humidified, 37°C, 5% CO_2_/95% air environment for 1 hr. Then, 5 × 10^4^ cells in various media were added and cultured in a 37°C, 5% CO_2_/95% air environment for 24 hrs. At that time, four random areas were visualized using light microscopy, photographed and analysed for the presence of tubular‐like structures. The total length of the formed tubular‐like formation was measured using commercially available ImageJ software.

### 3‐(4,5‐dimethylthiazol‐2‐yl)‐2,5‐diphenyltetrazolium bromide (MTT) assay

HUVECs were seeded at 10^4^ cells per each well of 96‐well plates in various media (containing 150 μM of H_2_O_2)_ in a humidified, 37°C, 5% CO_2_/95% air environment for 90 min. At that time, the cells were rinsed with PBS, and the MTT assay (Life Technologies) was performed. The results provided evidence regarding cell viability after challenge by H_2_O_2_.

### ROS measurement

To measure reactive oxygen species (ROS) production in HUVECs, DCFH‐DA, an oxidation‐sensitive indicator, was used. In the presence of ROS, DCFH is oxidized to fluorescent 2′,7′‐dichlorofluorescein (DCF), which can be measured by fluorometry. For this purpose, HUVECs were seeded at 10^4^ cells/well in 96‐well cell culture plates. The supernatant medium was removed, and DCFH‐DA was added at a concentration of 10 μM per well. These specimens were kept at 37°C for 1 hr. At that time and after rinsing the cells with PBS, CM from BMMSCs of either diabetic or control rats was added. Oxidative stress was induced using tert‐butyl hydroperoxide (Luperox, t‐BuOOH Sigma‐Aldrich (St. Louis, MO, USA) at 100 μM. Fluorescence intensity of the samples was measured using a fluorescence spectrophotometer at excitation and emissions wavelengths of 485 nm and 530 nm, respectively.

### RNA extraction, reverse transcription and quantitative polymerase chain reaction (qRT‐PCR)

Expression of select angiogenic markers by BMMSCs was determined using total RNA that had been extracted from these cells using TRIzol reagent (Life Technologies) according to the manufacturer's protocols. Real‐time PCR was performed using an iCycler thermocycling apparatus (MyiQ^™^ Single‐Color Real‐Time PCR, Bio‐Rad Laboratories, Marnes‐la‐Coquette, France). After activation of the DNA polymerase at 95°C for 10 min., cDNA was amplified by performing 40 two‐step PCR cycles: a 15 sec. denaturation step at 95°C, followed by a 60 sec. annealing and an extension step at 60°C. The Multiplex used contained 84 angiogenic‐related genes (the rat angiogenesis RT² Profiler PCR Array, Qiagen).

Each sample was run in triplicate, and each analysis was repeated on four separate occasions. Data were analysed using MyiQ^™^ Software (Bio‐Rad Laboratories). The data were normalized with respect to results obtained from LEAN‐BMMSCs.

### ELISA analysis

The level of 7 mediators (VEGF, IL‐6, TNFα, MCP‐1, FGF‐2, IGF1 and PLAU) were determined in the respective supernatant media at each time‐point of interest to this study using different ELISA kits and following the manufacturer's instructions. These experiments were conducted in triplicate and repeated on three separate occasions. A five‐parameter regression formula was used to calculate concentrations of the respective mediators using standard curves for each mediator assessed.

### Proteomic analysis

Each supernatant CM from LEAN and ZDF‐BMMSCs was separately concentrated and desalted using an Amicon Ultra‐2 Centrifugal device column (Millipore, France). Briefly, 500 μl of each conditioned medium tested was loaded in a column and centrifuged at 4500 × g for 75 min. This treatment resulted in a 10‐fold increase in protein concentration. The proteins in the concentrated CM were then analysed using a mass spectrometer (LTQ‐Orbitrap, Thermo Scientific, Waltham, MA, USA) at the Jacques‐Monod Institute (Paris, France). The data were normalized with respect to results obtained from LEAN‐BMMSCs.

### *In vivo* experiments

#### Matrigel plug assay

Samples of supernatant CM from either LEAN‐BMMSCs or ZDF‐BMMSCs, in αMEM either supplemented with 10% foetal calf serum or serum‐free, were freeze‐dried using a Christ Alpha 1‐2 freezer‐drier overnight. At that time, 500 μg protein from each dried CM sample was mixed with 500 μl of ice‐cold Matrigel (growth factor reduced Matrigel, VWR, France) and was injected subcutaneously into the flanks of 8‐week‐old Swiss nude mice 19. After 14 days, the mice were killed, and each Matrigel implant was excised, placed in 500 μl of RIPA buffer, and processed using a Retsch MM 300 tissue lyser at 30 pulses/min. for 2 min. The haemoglobin content in each excised implant was determined using a commercially available assay (Hemoglobin Colorimetric Assay kit, Cayman Ann Arbor, MI, USA). Plugs were also embedded in paraffin, sectioned, and stained with Masson's trichrome.

### Statistical analysis

Each experiment of this study was performed in triplicate and repeated with three independent experiments using different cell preparations. Numerical data are reported as the mean ± standard error of the mean (SEM). Student's *t*‐tests for unpaired two‐tailed samples were used for statistical analyses of the experimental data and compared to the respective controls. *P* value of < 0.05 was considered statistically significant.

## Results

### CM from BMMSCs of diabetic rats affects HUVEC functions

To characterize the impact of diabetes type 2 (T2DM) on the BMMSC secretome, the effect of CM on selected functions (specifically migration, proliferation, viability and reactive oxygen species production) of HUVECs as well as *in vitro* and *in vivo* angiogenesis was examined.

### CM from BMMSCs of diabetic rats increases HUVEC vascular‐like tube formation *in vitro*

**Figure 1:**
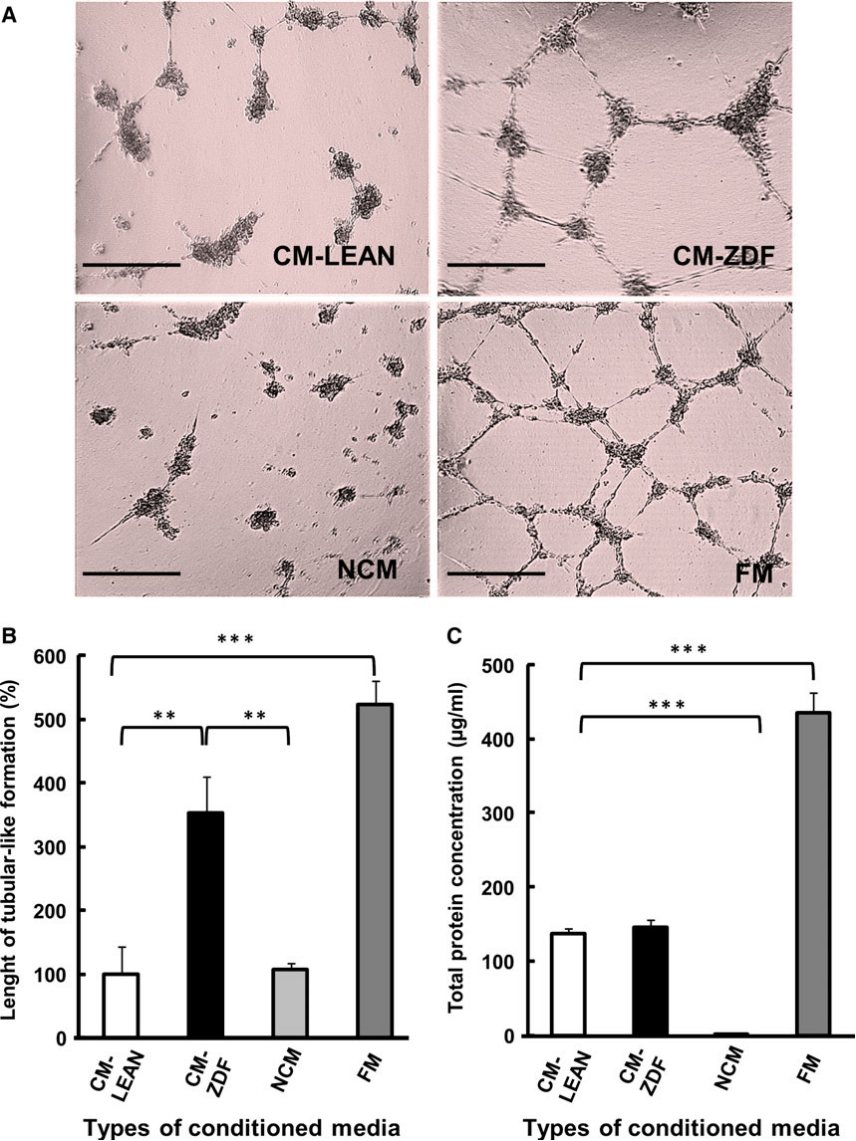
Conditioned supernatant medium from BMMSCs of diabetic rats promotes tubular‐like formation by HUVECs. (**A**) Representative images of HUVECs in CM‐LEAN, CM‐ZDF, FM or NCM cultured on Matrigel for 24 hrs showing formation of tubular‐like structures. Scale bar = 100 μm. (**B**) Quantification of the tubular‐like structures formed by HUVECs in CM‐LEAN, CM‐ZDF, FM or NCM cultured on Matrigel. Data are expressed as a percentage of the results obtained with CM‐LEAN (group control). (**C**) Protein content in the supernatant CM‐LEAN, CM‐ZDF, FM and NCM. Values are mean ± SEM of four randomly selected fields on each Matrigel surface tested. **P < 0.01, ***P < 0.001. CM‐LEAN: supernatant, conditioned medium from BMMSCs of control rats. CM‐ZDF: supernatant, conditioned medium from BMMSCs of diabetic rats. FM: Alpha MEM containing 10% foetal bovine serum (FBS) not exposed to cells. NCM: FBS‐free alpha MEM not exposed to cells.

The angiogenic potential of CM collected from either ZDF‐BMMSCs (CM‐ZDF) or LEAN‐BMMSCs (CM‐LEAN) was determined using HUVECs on Matrigel in the presence of CM‐ZDF, CM‐LEAN, αMEM containing 10% FBS (FM) or FBS‐free αMEM (NCM) for 24 hrs (Fig. 1A). The CM‐LEAN group was the reference in this experiment. The supernatant CM‐ZDF induced a significantly (*P* < 0.01) increased (3.5‐fold) formation of HUVEC tubular‐like structures compared to results obtained with either supernatant CM‐LEAN or NCM (Fig. 1A and B). The protein content in the CM‐LEAN and CM‐ZDF was similar (Fig. 1C), but lower (*P* < 0.01) than that in the FM (Fig. 1C). These results suggest that MSCs from diabetic rats secrete soluble, bioactive, angiogenic mediators into their supernatant medium that affect HUVEC tube‐like formation.

### CM from BMMSCs of diabetic rats promoted increased HUVEC migration *in vitro*

**Figure 2:**
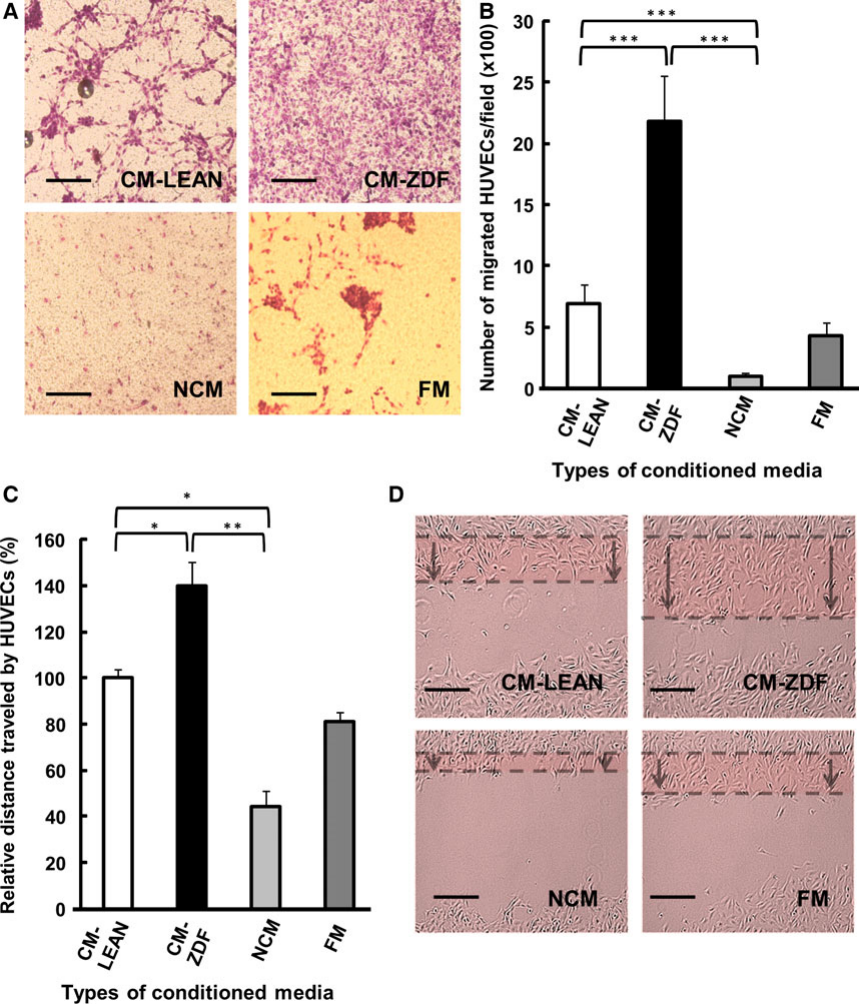
Conditioned supernatant medium from BMMSCs of diabetic rats induces increased HUVEC migration. HUVEC migration was determined using Boyden chambers. (**A**) Representative images showing HUVEC migration. Stain: Giemsa. Scale bar = 200 μm. (**B**) Number of migrated HUVECs in the presence of CM‐LEAN, CM‐ZDF, FM or NCM. HUVECs using the *in vitro* scratch wound‐healing assay. (**C**) Distance of migrated HUVECs exposed to supernatant CM‐LEAN, CM‐ZDF, FM or NCM. (**D**) Representative light micrographs of the scratch area after 6 hrs. The dotted lines delineate the original scratched area after 6 hrs. Scale bar = 100 μm. Data are expressed as a percentage of the results obtained from the control (CM‐LEAN) group. Values are mean ± SEM of four fields on each surface tested. *P < 0.05, **P < 0.01, ***P < 0.001. CM‐LEAN: supernatant, conditioned medium from BMMSCs of control rats. CM‐ZDF: supernatant, conditioned medium from BMMSCs of diabetic rats. FM: alpha MEM containing 10% foetal bovine serum (FBS), not exposed to cells. NCM: FBS‐free alpha MEM not exposed to cells.

The chemotactic potential of CM was examined using both the transwell cell migration assay and the *in vitro* scratch wound‐healing assay. HUVEC migration through the porous transwell membrane (8 μm pore diameter) was observed after 24 hrs with CM‐LEAN, CM‐ZDF or FM, but not when NCM was used as the chemoattractant (Fig. 2A). Compared with either CM‐LEAN or FM, CM‐ZDF induced significantly (*P* < 0.001) increased (3‐fold) migration of HUVECs (Fig. 2B). After 6 hrs, HUVEC migration in the scratch wound‐healing assay was significantly (*P* < 0.01) enhanced (1.4‐fold) in the presence of CM‐ZDF compared to results obtained with either CM‐LEAN or FM (Fig. 2C and D). These results provide evidence that BMMSCs from diabetic rats secrete soluble, bioactive, chemotactic mediators into their supernatant media, which promote HUVEC migration.

### CM from BMMSCs of diabetic rats does not affect either HUVEC proliferation or viability and has a limited effect on reactive oxygen species production

**Figure 3:**
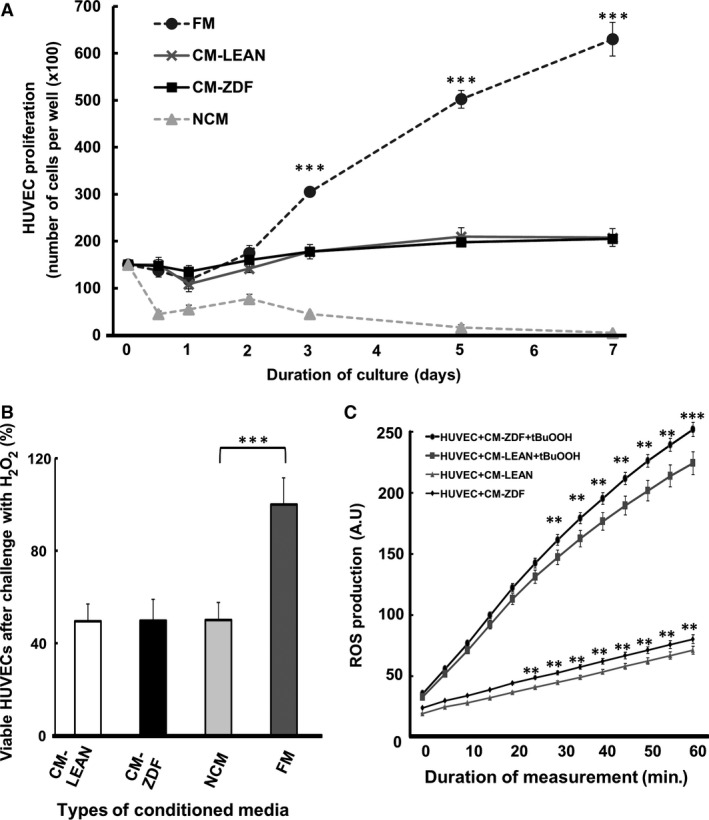
Conditioned supernatant medium from BMMSCs of either diabetic or healthy rats has no effect on either HUVEC proliferation or viability when challenged by H_2_O_2_, and a limited effect on reactive oxygen species production. (**A**) Proliferation of HUVECs in the presence of supernatant CM‐LEAN, CM‐ZDF, FM and NCM cultured for up to 7 days. (**B**) HUVEC apoptosis determined using the MTT assay. HUVECs were exposed to 150 μM of H_2_O_2_ in supernatant CM‐LEAN, CM‐ZDF, FM or NCM for 90 min. (**C**) ROS production by HUVECs exposed to either CM‐LEAN or CM‐ZDF for 5 hrs. A.U.: arbitrary units. Values are mean ± SEM. ***P < 0.001. CM‐LEAN: supernatant, conditioned medium from BMMSCs of control rats. CM‐ZDF: supernatant, conditioned medium from BMMSCs of diabetic rats. FM: alpha MEM containing 10% foetal bovine serum (FBS) not exposed to cells. NCM: FBS‐free alpha MEM not exposed to cells.

The effect of the supernatant CM collected from either ZDF‐BMMSCs or LEAN‐BMMSCs after 24 hrs of culture on the proliferation of HUVECs was assessed. For this purpose, HUVECs were cultured in CM‐LEAN, CM‐ ZDF, FM or NCM, and their proliferation was determined over a period of up to 7 consecutive days. Compared to results obtained from the other conditions tested, HUVECs cultured in FM exhibited increased proliferation starting at day 3; this trend increased by 4‐fold at day 7 of culture (Fig. 3A). The HUVECs did not survive in the absence of serum in the supernatant media (Fig. 3A). HUVECs cultured with either CM‐LEAN or CM‐ZDF survived but did not proliferate over the 7 days of the experiments. Moreover, the number of HUVECs under either CM‐ZDF or CM‐LEAN was similar for the duration of the experiments (Fig. 3A). The effect of the various supernatant CM on HUVEC viability after challenge with H_2_O_2_ was assessed by exposing HUVECs to 150 μM of H_2_O_2_ in each medium of interest to this study for 90 min. HUVECs exposed to CM‐LEAN, CM‐ZDF or NCM had similar numbers of cells, which were significantly (*P* < 0.001) higher than those observed under FM (Fig. 3B).

Furthermore, upon exposure to either CM‐LEAN or CM‐ZDF, production of ROS by HUVECs was higher when the cells were exposed to CM‐ZDF than to CM‐LEAN; this difference in ROS was approximately 15% after 1 hr of measurement (*P* < 0.001) (Fig. 3C).

### CM from BMMSCs of diabetic rats promotes increased neoangiogenesis *in vivo*

**Figure 4:**
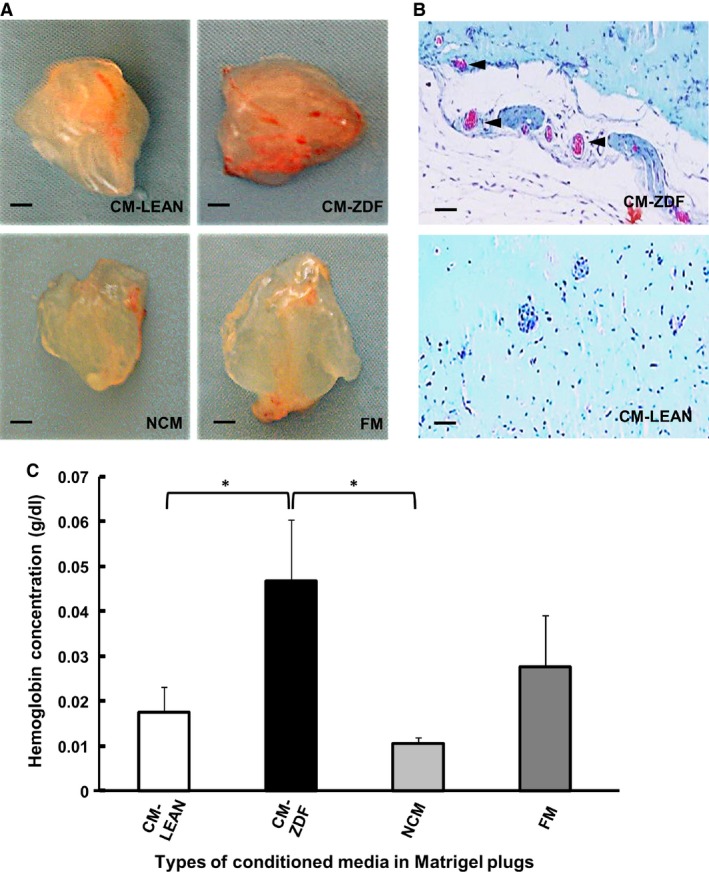
Conditioned supernatant medium from BMMSCs of diabetic rats enhances *in vivo* angiogenesis. Matrigel plugs (500 μl) containing proteins (500 μg) from freeze‐dried supernatant CM‐ZDF, CM‐LEAN, FM or NCM were injected subcutaneously into the flanks of nude mice. After 14 days, these Matrigel plugs were excised and photographed, and their haemoglobin contents were quantified. **(A)** Representative photographs of the excised Matrigel plugs. Scale bar = 1 mm. **(B)** Representative photograph of sectioned paraffin‐embedded Matrigel plugs stained with Masson's trichrome (the arrowhead indicates capillaries with red blood cells). Scale bar = 20 μm **(C)** Quantification of the haemoglobin contents in the excised Matrigel plugs. Values are mean ± SEM;* n* = 6 per experimental group. *P < 0.05. CM‐LEAN: supernatant, conditioned medium from BMMSCs of control rats. CM‐ZDF: supernatant, conditioned medium from BMMSCs of diabetic rats. FM: alpha MEM containing 10% FBS not exposed to cells. NCM: FBS‐free alpha MEM not exposed to cells.

To evaluate the proangiogenic potential of the CM‐ZDF *in vivo*, Matrigel plugs were implanted in a mouse model. These Matrigel plugs, prepared using either CM‐ZDF or CM‐LEAN (each containing 500 μg/ml of proteins), as well as either NCM or FM, were injected into the flanks of nude mice and were implanted for a 14‐day period. At that time, the explanted plugs containing NCM, CM‐LEAN or FM were pale in colour, indicating no or little blood vessel content (Fig. 4A). In contrast, plugs containing CM‐ZDF appeared dark red due to the presence of red blood cells, indicating the formation of blood vessels (Fig. 4A and B). Compared to plugs prepared with either CM‐LEAN or NCM, the implanted Matrigel plugs containing CM‐ZDF had a significant (***P*** < 0.05) increase (2.5‐fold) in haemoglobin content (Fig. 4C). In conjunction with the *in vitro* results, these *in vivo* data provided evidence that supernatant CM from ZDF‐BMMSCs promotes increased angiogenesis *in vivo*.

### BMMSCs of diabetic rats differentially express angiogenic genes

**Figure 5:**
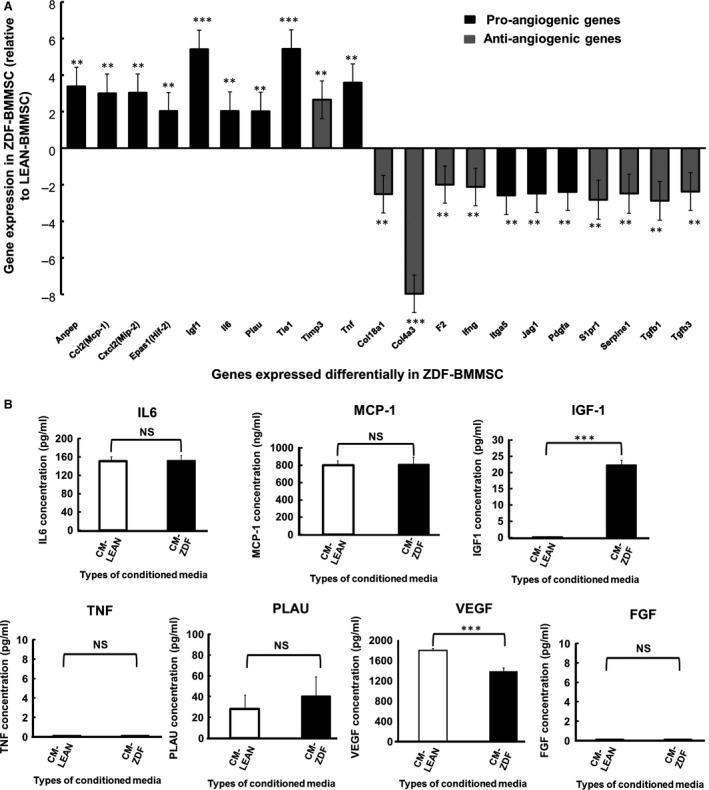
Expression of angiogenic gene mediators is modified in BMMSCs of diabetic rats. (**A**) Expression of select pro‐angiogenic genes (black bars) and anti‐angiogenic genes (grey bars) in ZDF‐MSCs relative to results obtained from LEAN‐MSsC. (**B**) ELISA results of the analysis of overexpressed pro‐angiogenic genes in samples from ZDF‐MSCs. Values are mean ± SEM. *n* = 3. ***P < 0.001. LEAN‐MSCs: BMMSCs from control rats. ZDF‐MSC: BMMSCs from diabetic rats. CM‐LEAN: supernatant, conditioned medium from BMMSCs of control rats. CM‐ZDF: supernatant conditioned medium from BMMSCs of diabetic rats.

The underlying mechanism(s) of the observed increased angiogenesis of the CM‐ZDF was explored by determining the expression of 84 known angiogenic genes in BMMSCs. Nine pro‐angiogenic genes, including alanyl aminopeptidase (ANPEP) (3‐fold), monocyte chemoattractant protein‐1 (MCP‐1) (3‐fold), macrophage inflammatory protein 2‐alpha (MIP‐2) (3‐fold), hypoxia‐inducible factor 2 (HIF‐2) (2‐fold), IGF‐1 (5.5‐fold), interleukin 6 (IL‐6) (2‐fold), urokinase‐type plasminogen activator (PLAU) (2‐fold), tyrosine kinase with immunoglobulin‐like and EGF‐like domains 1 (TIE1) (5.5‐fold) and tumour necrosis factor alpha (TNFα) (4‐fold), were significantly (*P* < 0.05) up‐regulated in ZDF‐BMMSCs. In addition, eight anti‐angiogenic genes, including collagen type XVIII alpha 1 (COL18A1) (2‐fold), collagen type IV alpha 3 (COL4A3) (8‐fold), coagulation Factor II (F2) (2‐fold), interferon gamma (INFγ) (2‐fold), Sphingosine‐1‐phosphate receptor 1 (S1PR1) (3‐fold), Serpine1 (3‐fold), transforming growth factor beta 1 (TGFβ1) (3‐fold) and transforming growth factor beta 3 (TGFβ3) (2‐fold), were down‐regulated in ZDF‐BMMSCs (Fig. 5A). Several other angiogenic mediators, including vascular endothelial growth factor alpha (VEGFα), fibroblast growth factor 2 (FGF2), epidermal growth factor (EGF) and hypoxia‐inducible factor 1 alpha (HIF1α), were expressed similarly by both ZDF‐BMMSCs and LEAN‐BMMSCs (data not shown).

**Figure 6:**
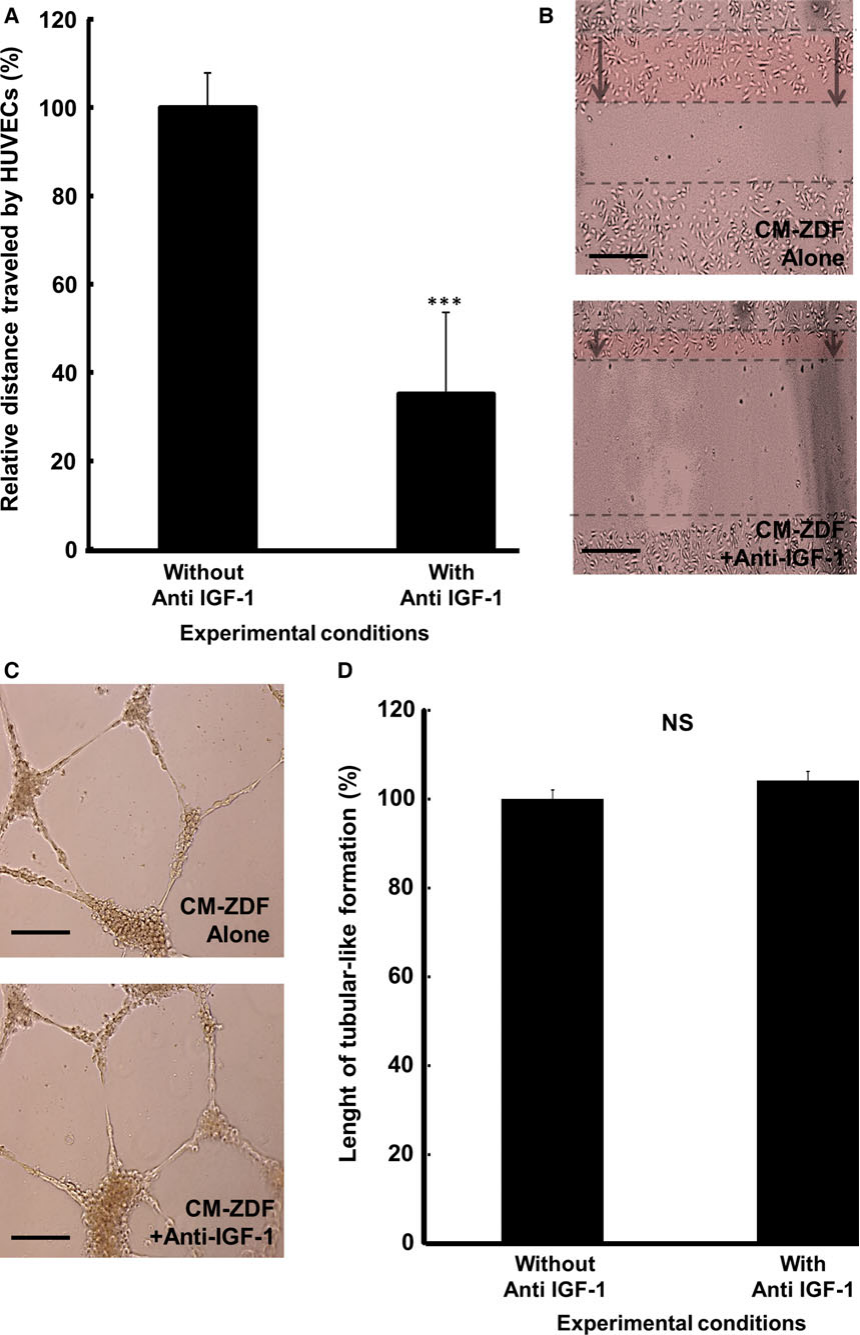
IGF‐1 in CM‐ZDF promotes migration of HUVECs but not tubular formation. HUVEC migration assays were performed using the *in vitro* scratch/wound‐healing assay. (**A**) Distance of migrated HUVECs exposed to either supernatant CM‐ZDF or CM‐ZDF containing a blocking antibody for IGF‐1. (**B**) Representative light micrographs of the scratch area after 6 hrs. The dotted lines delineate the original scratched area after 6 hrs. Scale bar = 100 μm. (**C**) Representative images of HUVECs in either CM‐ZDF without or CM‐ZDF with a blocking antibody for IGF‐1 (anti‐IGF‐1) cultured on Matrigel for 24 hrs. Scale bar = 100 μm. (**D**) Quantification of the tubular‐like structures formed by HUVECs in either CM‐ZDF without or CM‐ZDF with blocking antibody for IGF‐1 cultured on Matrigel. Data are expressed as a percentage of the results obtained with CM‐ZDF without anti‐IGF‐1 (control group). Values are mean ± SEM of four fields on each surface tested. ***P < 0.001.

ELISA analysis of the protein content in the conditioned media provided evidence that among the five different mediators tested, only the content of IGF‐1 correlated (*P* < 0.001) with the observed difference in gene expression due to ZDF‐BMMSCs (Fig. 5B). The concentrations of IL‐6, MCP‐1 and PLAU were similar in CM from LEAN‐ and ZDF‐BMMSCs. TNFα and FGF were not detected in all media tested (Fig. 5B), while the VEGF concentration was significantly (*P* < 0.001) lower in ZDF‐BMMSCs. The presence of bioactive IGF‐1 in CM from ZDF‐BMMSCs was confirmed by the reduced (by 60%; *P* < 0.001) HUVEC migration when this CM was supplemented with antibodies against IGF‐1 (Fig. 6A and B). Blocking IGF‐1 in CM from ZDF‐BMMSCs did not affect the formation of tubular structures (Fig. 6C and D).

### CM from BMMSCs of diabetic rats overexpressed several extracellular matrix proteins related to angiogenesis

**Figure 7:**
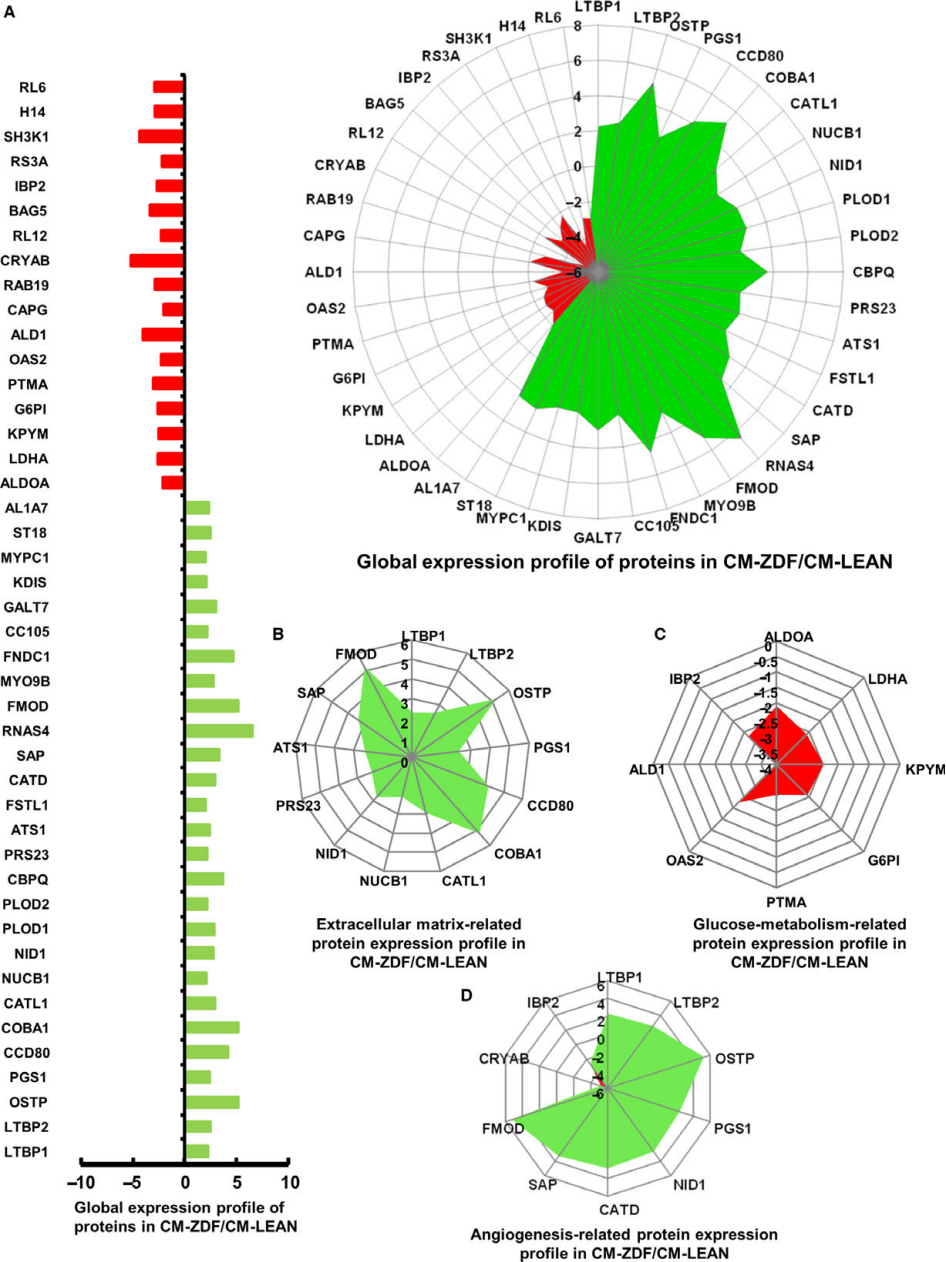
The content of angiogenic mediators in conditioned medium (CM) from BMMSCs of diabetic rats and of controls is different Proteomic analysis of the protein content in supernatant CM‐ZDF compared to the results obtained from CM‐LEAN. Down‐regulated and overexpressed proteins are shown in red and green, respectively, in all frames. (**A**) Proteins that were differentially expressed in supernatant CM‐ZDF compared to CM‐LEAN (**B**) Proteins present in the supernatant CM‐ZDF that are related to the extracellular matrix. (**C**) Proteins present in the supernatant CM‐ZDF that are related to glucose metabolism. (**D**) Proteins present in the supernatant CM‐ZDF that are related to angiogenesis. Values are mean ± SEM. *n* = 3. *P* < 0.05. CM‐LEAN: supernatant, conditioned medium from BMMSCs of control rats. CM‐ZDF: supernatant, conditioned medium from BMMSCs of diabetic rats.

Proteomic analysis of the supernatant CM from either diabetic or control BMMSCs revealed over 2‐fold (*P* < 0.05) up‐regulation of 27 and down‐regulation of 17 proteins in the CM‐ZDF (Fig. 7A). Eight proteins related to glucose metabolism, specifically aldolase, fructose‐bisphosphate A (ALDOA) (by 2‐fold), lactate dehydrogenase A (LDHA) (by 2.5‐fold), pyruvate kinase, muscle (KPYM) (by 2.5‐fold), glucose‐6‐phosphate (G6P) (by 2.5‐fold), prothymosin, alpha (PTMA) (by 3‐fold), 2′‐5′‐oligoadenylate synthetase 2 (OAS2) (by 2‐fold), aldehyde dehydrogenase 1 (ALD1) (by 4‐fold) and insulin‐like growth factor binding protein 2 (IBP2) (by 2.5‐fold), were differentially expressed and down‐regulated in the CM of ZDF‐BMMSCs (Fig. 7B). This result indicates the possible modification of glucose metabolism in the BMMSCs from diabetic rats.

Several proteins whose genes were overexpressed by the ZDF‐BMMSCs are either extracellular matrix components or are involved in the process of extracellular matrix remodelling (Fig. 7C), including latent transforming growth factor beta binding protein 1 (LTBP1) (2‐fold), latent transforming growth factor beta binding protein 2 (LTBP2) (2‐fold), osteopontin (OSTP) (5‐fold), phosphatidylglycerophosphate synthase 1 (PGS1) (2‐fold), coiled‐coil domain containing 80 (CCD80) (4‐fold), alamin adenosyltransferase (COBA1) (5‐fold), cathepsin L1 (CATL1) (2‐fold), nucleobindin 1 (NUCB1) (2‐fold), nidogen 1 (NID1) (2‐fold), serine protease 23 (PRS23) (2‐fold), ADAM metallopeptidase with thrombospondin type 1 motif, 1(ATS1) (2‐fold), serum amyloid P (SAP) (3‐fold) and fibromodulin (FMOD) (5‐fold).

**Figure 8:**
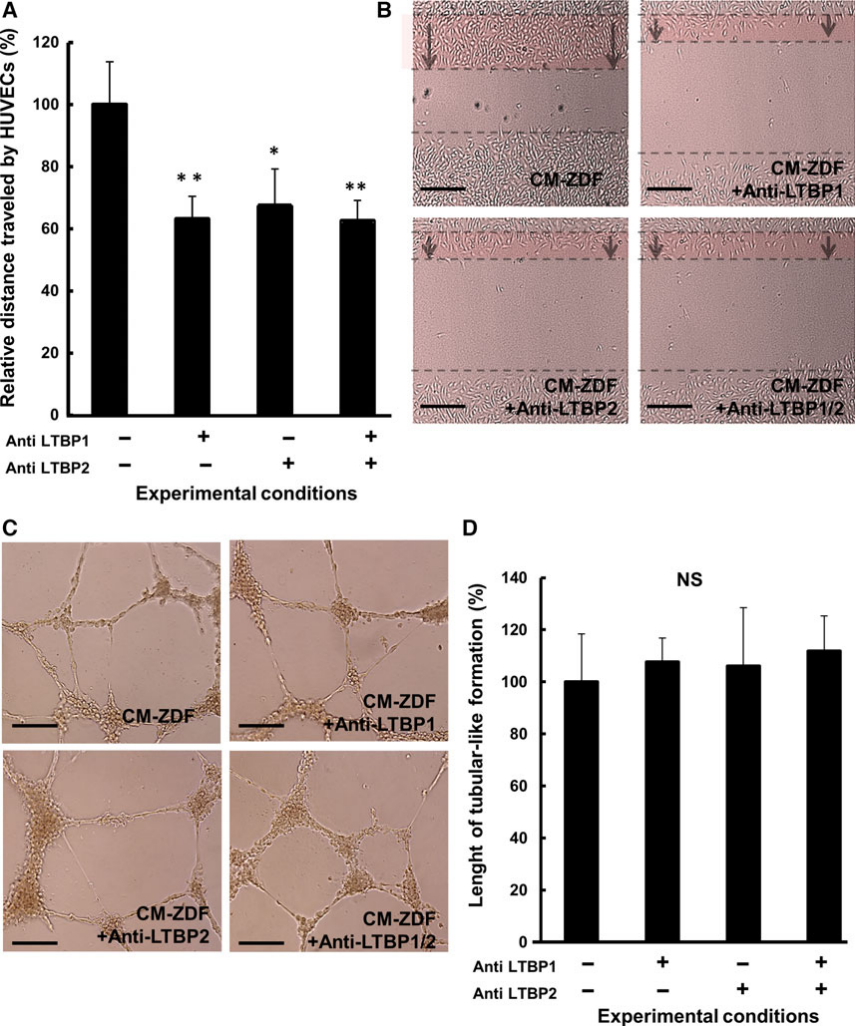
Specific extracellular matrix proteins (LTBP‐1 and LTBP‐2) are involved in the enhanced migration of CM‐ZDF. HUVEC migration assays were performed using the *in vitro* scratch/wound‐healing assay. (**A**) Distances migrated by HUVECs exposed to either supernatant CM‐ZDF or CM‐ZDF in the presence or absence of blocking antibodies for either LTBP‐1 or LTBP‐2 or both. (**B**) Representative light micrographs of the scratch area 6 hrs post cell injury. The dotted lines delineate the original scratched area after 6 hrs. Scale bar = 100 μm. (**C**) Representative images of HUVECs in either CM‐ZDF or CM‐ZDF and in the presence or absence of blocking antibodies for LTBP‐1, LTBP‐2 or both, cultured on Matrigel for 24 hrs. Scale bar = 100 μm. (**D**) Quantification of the tubular‐like structures formed by HUVECs exposed to either CM‐ZDF or CM‐ZDF in the presence or absence of blocking antibodies for LTBP‐1, LTBP‐2, or both, cultured on Matrigel for 24 hrs. Data are expressed as percentage of the results obtained when the cells were cultured in CM‐ZDF (control group). Values are mean ± SEM of four fields on each surface tested. *P < 0.05, **P < 0.01.

Some of these components could be related to angiogenesis and may be involved in the observed enhanced chemotactic response of endothelial cells (Fig. 7D). In fact, among the proteins differentially expressed in ZDF‐BMMSC CM, seven proteins, specifically LTBP1 (2‐fold), LTBP2 (2‐fold), OSTP (5‐fold), cathepsin D (CATD) (2‐fold), SAP (2‐fold) and FMOD (5‐fold), are related to angiogenesis and were overexpressed (Fig. 7D). Blocking either LTBP1 or LTBP2 in the CM from ZDF‐BMMSCs resulted in a decrease (by 40%) of the stimulatory effect of these chemical compounds on HUVEC migration (Fig. 8A and B) but did not affect tubular structure formation (Fig. 8C and D). These results indicate that compared to the secretome of control BMMSCs, the secretome of ZDF‐BMMSCs contains matrix‐related proteins (such as LTBP1 or LTBP2) and promotes angiogenesis (Fig. 8).

## Discussion

Despite the fact that BMMSC‐derived paracrine factors were reported to be largely involved in neovascularization and tissue repair, the impact of type 2 diabetes mellitus (T2DM) on the BMMSC secretome and its effects on angiogenesis are still unknown 16, 17. We, therefore, performed a comparative analysis of the BMMSC secretome and its effects on endothelial cells, using ZDF rats at 13 weeks to represent early diabetic conditions and their LEAN littermates as controls. The results obtained provided the first evidence that short‐term T2DM alters the BMMSC secretome composition and promotes angiogenic capabilities.

The results of this study demonstrated that angiogenesis‐related genes are differently expressed in BMMSCs from ZDF rats when compared with results from LEAN animals. In fact, several pro‐angiogenic genes were overexpressed, while anti‐angiogenic genes were down‐regulated (Fig. 5). The up‐regulated (over 5‐fold) genes included IGF‐1 and TIE1, which are critical regulators of angiogenesis 20, MCP‐1 and IL‐6, homing factors for BMMSCs and EC/EPCs, respectively 21, 22, and IL‐6 and TNFα, critical mediators of the inflammatory process. Nevertheless, the expression of several angiogenic genes did not correlate with the released protein levels in the supernatant CM in this study. Analysis of the protein content in the 24‐hr secreted CM from LEAN‐ and ZDF‐BMMSCs provided evidence that among the different mediators tested, only the content of IGF‐1 correlated (*P* < 0.001) with the observed difference in gene expression by ZDF‐BMMSCs (Fig. 5B). However, proteomic analysis did not reveal IGF1 in the CM. This imperfect and variable correlation between ELISA and proteomic analysis can be explained by different levels of sensitivity and dynamic ranges of the methods 23. The concentrations of IL‐6, MCP‐1 and PLAU were similar in CM from LEAN‐ and ZDF‐BMMSCs, while that of VEGF was lower in ZDF‐BMMSCs. TNFα and FGF were not detected in all media tested. IGF‐1 plays a significant role in the physiology of endothelial cells by promoting their migration 20, 24. IGF‐1 is also overexpressed by several types of cells implicated in T2DM‐proliferative retinopathy 25, 26. Under our experimental conditions, IGF‐1 released by ZDF‐BMMSCs accounted for approximately 60% of the observed enhanced HUVEC migration (Fig. 6A and B). The IGF‐1 effect is mediated by activation of the IGF‐1 receptor, which triggers several signalling pathways and therefore several cellular responses. For instance, the Ras/Raf pathway is critical for proliferative responses, whereas activation of Rac is important for cell migration 27. This study provides evidence that IGF‐1 might be a key factor in the paracrine action of T2DM‐BMMSCs on endothelial cell behaviour, thus contributing to the pathogenesis of microvascular complications associated with the diabetic condition, including diabetic retinopathy.

Moreover, proteomic analysis of the T2DM‐BMMSC secretome revealed some proteins that are related to angiogenesis through several mechanisms. Specifically, the results showed (i) decreased levels of αβ‐crystallin, a chaperone for VEGF‐A, and (ii) increased levels of LTBP1 and LTBP2, regulators of TGF‐β availability 28, as well as of OSTP and FMOD, which are components of the extracellular matrix and might be involved in the paracrine action of T2DM‐BMMSCs on endothelial cells 29, 30, 31, 32. The role of extracellular matrix components in angiogenesis has been highlighted by both *in vitro* and *in vivo* studies 33, 34. In this respect, this study revealed a different secretory profile of BMMSCs that may explain how these cells affect the environmental niche and paradoxical angiogenesis in the diabetic milieu. ROS stands at the cross‐point of the regulations exerted on the BMMSCs as a signalling molecule in the bone marrow environment 35. For instance, low levels of ROS are associated with quiescent MSCs, whereas an increased ROS level is typical of proliferating cultures.

In addition, the proteomic analysis of T2DM‐BMMSC CM demonstrated a specific secretory phenotype of extracellular matrix remodelling and glucose metabolism. Compared to LEAN‐BMMSCs, of the 261 proteins released by ZDF‐BMMSCs, 44 were differentially secreted, with 27 at higher and 17 at lower levels (Fig. 7). The 27 overexpressed proteins included extracellular matrix homeostasis and remodelling‐related molecules 36. In contrast, proteins involved in the metabolism of glucose (such as ALDOA, LDHA, KPYM, G6P, PTMA, OAS2, ALD1 and IBP2) were secreted at lower levels (Fig. 7C). Impaired glucose metabolism was already observed in T2DM muscle cells 37, but to our knowledge, this is the first report in the BMMSC secretome. Targeting such molecules may offer insight into the underlying mechanisms of T2DM‐BMMSC physiology.

Compared with LEAN‐BMMSCs, the ZDF‐BMMSC secretome was unique, despite the fact that ZDF‐BMMSCs were extensively expanded by *ex vivo* culture at normal glucose levels. These findings show that the effects of the diabetic milieu were evident on the BMMSCs, even after multiple cell divisions *in vitro* in a normoglycaemic environment. Moreover, these results corroborate literature reports that aspects of the diabetic condition, particularly hyperglycaemia, induce transmissible alterations in many type of cells, including endothelial, vascular smooth muscle, retinal and cardiac cells 38. In fact, the process by which hypo‐methylation of gene promoters leads to deregulated gene expression in T2DM is known as ‘metabolic memory’ 38, 39 and is implicated in the persistence of vascular complications of diabetes, even after return to normal blood glucose levels 38, 39. Regarding ZDF rats, alteration of hepatic DNA methylation at early diabetic conditions (12 weeks) was previously reported 40. For this reason, it is possible that the altered secretome observed in ZDF‐BMMSCs is due to a ‘metabolic memory’ phenomenon. Further studies are needed to determine whether such changes are initiated during the pre‐diabetic state in the ZDF rat model.

This study is the first to directly compare both *in vitro* and *in vivo* the angiogenic potential of BMMSC‐CM from T2DM and control animals. In line with previous studies reporting the angiogenic potential of the BMMSC secretome 17, 41, BMMSC‐CM from both, ZDF rats and age‐matched LEAN rats also showed angiogenic properties. These results corroborated previous studies establishing that healthy rat BMMSCs and CM‐BMMSC improve the formation of HUVEC tubular structure in both paracrine 41, 42 and juxtacrine manners 41. The CM from BMMSCs, however, affected endothelial cell functions differently. It promoted HUVEC migration and tube formation with no change in proliferation. Such differences are due to changes in the levels of several bioactive compounds, such as IGF‐1, LTBP1 and LTBP2, and reflect the interactive and possible synergistic action of those multiple bioactive mediators leading to a promotion of angiogenesis. Another plausible explanation for the differences in effects of CM from BMMSCs on HUVEC functions may be the relative concentrations of paracrine factors. For instance, IGF‐1‐related effects are mediated by activation of IGF‐1 receptors, which trigger, among others, the Rac pathway. This pathway is important for cell migration but not proliferation 27. Our findings are in line with Gruber *et al*. 43 who suggested that MSCs paracrine effects might not be responsible for the initiation of the angiogenic process, but rather would play a role in guiding the growing blood vessels. However, in contrast with these data, Kinnaird *et al*. 17 reported that human BMMSCs MC increases EC proliferation. The reasons from these mixed and discrepant results are not really surprising if one takes into consideration the differences in isolation methods, species (rat *versus* human) and EC sources use 44. Moreover, in this study, CM from rat BMMSCs induced vascular formation in matrigel plugs *in vivo*. These findings are in line with previous studies 45 that show the occurrence of functional blood vessels in rBMMSC‐CM loaded Matrigel plugs. Taken together, these data indicate that BMMSCs secretome may contribute to EPCs recruitment and subsequent formation of functional vessels.

Another important result of this study is that the CM from T2DM–BMMSCs increased *in vitro* endothelial cell migration and tubular structure formation, as well as *in vivo* vascular formation in matrigel transplants (Fig. 4). The greater angiogenic potential of T2DM–BMMSCs secretome may reflect the interactive actions of multiple bioactive mediators (including IGF‐1, LTBP1 and LTBP2) which may alter the functions of endothelial cells, key players in diabetic vascular complications 46. Angiogenesis results from an equilibrium between pro‐ and anti‐angiogenic factors 46. The increased angiogenesis observed in this study may arise from a disturbed balance between these pro‐ and anti‐angiogenic factors and highlights the paracrine effect of an altered BMMSCs secretome on endothelial cells. It further illustrates the ambiguous role of angiogenesis in the pathogenesis of diabetic vascular complications. Thus, exacerbated angiogenesis occurs in diabetic retinopathy and nephropathy, whereas reduced angiogenesis contributes to diabetic impaired wound healing. A similar response to that observed in this study had been reported for BMMSCs cultured under *in vitro* hypoxic and high glucose conditions used to mimic the diabetic environment *in vitro* 47, 48. This increase in angiogenic response may reflect the ‘adaptive’ BMMSC response to the severe stress conditions of the diabetic milieu. Because CM delivers paracrine/trophic angiogenic factors, it is important to take these aspects of diabetic pathology into consideration in the development of BMMSC‐based therapies aimed at improving tissue repair in diabetes. Last but not least, the findings reported here are somewhat different from the results of Dzhoyashvili *et al*. 49 who have shown that CM from human diabetic adipose tissue exhibited a reduced angiogenic potential. These apparently contradictory data further illustrate the complexity the pathogenesis of diabetic vascular complications and deserve supplementary investigations to fully decipher the molecular basis of MSC paracrine effects in diabetes.

A limitation of this study is the fact that it is difficult to separate the effects of hyperglycaemia from those of obesity. The ZDF rat was chosen as the animal model because these animals initially develop insulin resistance followed by T2DM at predictable ages and display many of the human T2DM characteristics, including obesity, abnormal blood lipid profiles, and related vascular complications 18, making them a clinically relevant model.

In summary, this study provided the first evidence that compared with control animals, BMMSCs from T2DM rats exhibit an altered secretome pattern and angiogenic properties and underline the critical role of paracrine factors of MSC‐mediated angiogenesis in the diabetic milieu. These results provide valuable insight in the vascular complications associated with diabetes and could be used in the development of BMMSC‐based therapies to improve diabetic tissue repair.

## Author contributions

J.R.: data research, analysis, and interpretation and manuscript writing; G.C.: data analysis; J.P.: data interpretation; C.B‐V: data analysis, design and interpretation; H.P.: discussion and final approval of manuscript; F.A.: conception, design, data analysis and interpretation and manuscript writing.

## Conflict of interest

The authors declare that they have no conflicts of interest.

## References

[1] Martin A , Komada MR , Sane DC . Abnormal angiogenesis in diabetes mellitus. Med Res Rev. 2003; 23: 117–45. 12500286 10.1002/med.10024

[2] Oikawa A , Siragusa M , Quaini F , *et al* Diabetes mellitus induces bone marrow microangiopathy. Arterioscler Thromb Vasc Biol. 2010; 30: 498–508. 20042708 10.1161/ATVBAHA.109.200154 PMC3548136

[3] Tahergorabi Z , Khazaei M . Imbalance of angiogenesis in diabetic complications: the mechanisms. Int J Prev Med. 2012; 3: 827–38. 23272281 10.4103/2008-7802.104853 PMC3530300

[4] Abu El‐Asrar AM , Nawaz MI , Kangave D , *et al* Angiogenesis regulatory factors in the vitreous from patients with proliferative diabetic retinopathy. Acta Diabetol. 2013; 50: 545–51. 21947384 10.1007/s00592-011-0330-9

[5] Abu El‐Asrar AM , Struyf S , Kangave D , *et al* Chemokines in proliferative diabetic retinopathy and proliferative vitreoretinopathy. Eur Cytokine Netw. 2006; 17: 155–65. 17194635

[6] Golden SH . Emerging therapeutic approaches for the management of diabetes mellitus and macrovascular complications. Am J Cardiol. 2011; 108: 59B–67B. 10.1016/j.amjcard.2011.03.017 21802582

[7] Gealekman O , Brodsky SV , Zhang F , *et al* Endothelial dysfunction as a modifier of angiogenic response in Zucker diabetic fat rat: amelioration with Ebselen. Kidney Int. 2004; 66: 2337–47. 15569324 10.1111/j.1523-1755.2004.66035.x

[8] Shin L , Peterson DA . Impaired therapeutic capacity of autologous stem cells in a model of type 2 diabetes. Stem Cells Transl Med. 2012; 1: 125–35. 23197759 10.5966/sctm.2012-0031 PMC3659680

[9] Roberts AC , Porter KE . Cellular and molecular mechanisms of endothelial dysfunction in diabetes. Diabetes Vasc Dis Res. 2013; 10: 472–82. 10.1177/1479164113500680 24002671

[10] Sumpio BE , Riley JT , Dardik A . Cells in focus: endothelial cell. Int J Biochem Cell Biol. 2002; 34: 1508–12. 12379270 10.1016/s1357-2725(02)00075-4

[11] Capla JM , Grogan RH , Callaghan MJ , *et al* Diabetes impairs endothelial progenitor cell‐mediated blood vessel formation in response to hypoxia. Plast Reconstr Surg. 2007; 119: 59–70. 17255657 10.1097/01.prs.0000244830.16906.3f

[12] Kang L , Chen Q , Wang L , *et al* Decreased mobilization of endothelial progenitor cells contributes to impaired neovascularization in diabetes. Clin Exp Pharmacol Physiol. 2009; 36: e47–56. 19558529 10.1111/j.1440-1681.2009.05219.x

[13] Watt SM , Gullo F , van der Garde M , *et al* The angiogenic properties of mesenchymal stem/stromal cells and their therapeutic potential. Br Med Bull. 2013; 108: 25–53. 24152971 10.1093/bmb/ldt031 PMC3842875

[14] Yan J , Tie G , Wang S , *et al* Type 2 diabetes restricts multipotency of mesenchymal stem cells and impairs their capacity to augment postischemic neovascularization in db/db mice. J Am Heart Assoc. 2012; 1: e002238. 23316315 10.1161/JAHA.112.002238 PMC3540677

[15] Pacini S , Petrini I . Are MSCs angiogenic cells? New insights on human nestin‐positive bone marrow‐derived multipotent cells. Front Cell Dev Biol. 2014; 2: 20. 25364727 10.3389/fcell.2014.00020 PMC4207020

[16] Hung SC , Pochampally RR , Chen SC , *et al* Angiogenic effects of human multipotent stromal cell conditioned medium activate the PI3K‐Akt pathway in hypoxic endothelial cells to inhibit apoptosis, increase survival, and stimulate angiogenesis. Stem Cells. 2007; 25: 2363–70. 17540857 10.1634/stemcells.2006-0686

[17] Kinnaird T , Stabile E , Burnett MS , *et al* Marrow‐derived stromal cells express genes encoding a broad spectrum of arteriogenic cytokines and promote *in vitro* and *in vivo* arteriogenesis through paracrine mechanisms. Circ Res. 2004; 94: 678–85. 14739163 10.1161/01.RES.0000118601.37875.AC

[18] Etgen GJ , Oldham B . Profiling of Zucker diabetic fatty rats in their progression to the overt diabetic state. Metabolism. 2000; 49: 684–88. 10831184 10.1016/s0026-0495(00)80049-9

[19] Benslimane‐Ahmim Z , Heymann D , Dizier B , *et al* Osteoprotegerin, a new actor in vasculogenesis, stimulates endothelial colony‐forming cells properties. J Throm Haemost. 2011; 9: 834–43. 10.1111/j.1538-7836.2011.04207.x 21255246

[20] Shigematsu S , Yamauchi K , Nakajima K , *et al* IGF‐1 regulates migration and angiogenesis of human endothelial cells. Endocr J. 1999; 46(Suppl): S59–62. 12054122 10.1507/endocrj.46.suppl_s59

[21] Middleton K , Jones J , Lwin Z , *et al* Interleukin‐6: an angiogenic target in solid tumours. Crit Rev Oncol Hematol. 2014; 89: 129–39. 24029605 10.1016/j.critrevonc.2013.08.004

[22] Salcedo R , Ponce ML , Young HA , *et al* Human endothelial cells express CCR2 and respond to MCP‐1: direct role of MCP‐1 in angiogenesis and tumor progression. Blood. 2000; 96: 34–40. 10891427

[23] Ranganath SH , Levy O , Inamdar MS , *et al* Harnessing the mesenchymal stem cell secretome for the treatment of cardiovascular disease. Cell Stem Cell. 2012; 10: 244–58. 22385653 10.1016/j.stem.2012.02.005 PMC3294273

[24] Bach LA . Endothelial cells and the IGF system. J Mol Endocrinol. 2015; 54: R1–13. 25351818 10.1530/JME-14-0215

[25] Romaniuk D , Kimsa MW , Strzalka‐Mrozik B , *et al* Gene expression of IGF1, IGF1R, and IGFBP3 in epiretinal membranes of patients with proliferative diabetic retinopathy: preliminary study. Mediators Inflamm. 2013; 2013: 986217. 24379526 10.1155/2013/986217 PMC3863537

[26] Beltramo E , Lopatina T , Berrone E , *et al* Extracellular vesicles derived from mesenchymal stem cells induce features of diabetic retinopathy *in vitro* . Acta Diabetol. 2014; 51: 1055–64. 25374383 10.1007/s00592-014-0672-1

[27] Delafontaine P , Song YH , Li Y . Expression, regulation, and function of IGF‐1, IGF‐1R, and IGF‐1 binding proteins in blood vessels. Arterioscler Thromb Vasc Biol. 2004; 24: 435–44. 14604834 10.1161/01.ATV.0000105902.89459.09

[28] Tatti O , Vehvilainen P , Lehti K , *et al* MT1‐MMP releases latent TGF‐beta1 from endothelial cell extracellular matrix *via* proteolytic processing of LTBP‐1. Exp Cell Res. 2008; 314: 2501–14. 18602101 10.1016/j.yexcr.2008.05.018

[29] Todorovic V , Rifkin D . LTBPs, more than just an escort service. J Cell Biochem. 2012; 113: 410–8. 22223425 10.1002/jcb.23385 PMC3254144

[30] Kale S , Raja R , Thorat D , *et al* Osteopontin signaling upregulates cyclooxygenase‐2 expression in tumor‐associated macrophages leading to enhanced angiogenesis and melanoma growth *via* alpha9beta1 integrin. Oncogene. 2014; 33: 2295–306. 23728342 10.1038/onc.2013.184

[31] Berchem G , Glondu M , Gleizes M , *et al* Cathepsin‐D affects multiple tumor progression steps *in vivo*: proliferation, angiogenesis and apoptosis. Oncogene. 2002; 21: 5951–5. 12185597 10.1038/sj.onc.1205745

[32] Jian J , Zheng Z , Zhang K , *et al* Fibromodulin promoted *in vitro* and *in vivo* angiogenesis. Biochem Biophys Res Commun. 2013; 436: 530–5. 23770359 10.1016/j.bbrc.2013.06.005 PMC4007216

[33] Neve A , Cantatore FP , Maruotti N , *et al* Extracellular matrix modulates angiogenesis in physiological and pathological conditions. BioMed Res Int. 2014; 2014: 756078. 24949467 10.1155/2014/756078 PMC4052469

[34] Sottile J . Regulation of angiogenesis by extracellular matrix. Biochim Biophys Acta. 2004; 1654: 13–22. 14984764 10.1016/j.bbcan.2003.07.002

[35] Lyublinskaya OG , Borisov YG , Pugovkina NA , *et al* Reactive oxygen species are required for human mesenchymal stem cells to initiate proliferation after the quiescence exit. Oxid Med Cell Longev. 2015; 2015: 502105. 26273423 10.1155/2015/502105 PMC4530296

[36] Monnier VM , Mustata GT , Biemel KL , *et al* Cross‐linking of the extracellular matrix by the maillard reaction in aging and diabetes: an update on ‘a puzzle nearing resolution’. Ann N Y Acad Sci. 2005; 1043: 533–44. 16037276 10.1196/annals.1333.061

[37] Gao Y , Wu F , Zhou J , *et al* The H19/let‐7 double‐negative feedback loop contributes to glucose metabolism in muscle cells. Nucleic Acids Res. 2014; 42: 13799–811. 25399420 10.1093/nar/gku1160 PMC4267628

[38] Reddy MA , Zhang E , Natarajan R . Epigenetic mechanisms in diabetic complications and metabolic memory. Diabetologia. 2015; 58: 443–55. 25481708 10.1007/s00125-014-3462-y PMC4324095

[39] Park LK , Maione AG , Smith A , *et al* Genome‐wide DNA methylation analysis identifies a metabolic memory profile in patient‐derived diabetic foot ulcer fibroblasts. Epigenetics. 2014; 9: 1339–49. 25437049 10.4161/15592294.2014.967584 PMC4622843

[40] Williams KT , Schalinske KL . Tissue‐specific alterations of methyl group metabolism with DNA hypermethylation in the Zucker (type 2) diabetic fatty rat. Diabetes Metab Res Rev. 2012; 28: 123–31. 21818837 10.1002/dmrr.1281

[41] Mohammadi E , Nassiri SM , Rahbarghazi R , *et al* Endothelial juxtaposition of distinct adult stem cells activates angiogenesis signaling molecules in endothelial cells. Cell Tissue Res. 2015; 362: 597–609. 26068799 10.1007/s00441-015-2228-2

[42] Li H , Zuo S , He Z , *et al* Paracrine factors released by GATA‐4 overexpressed mesenchymal stem cells increase angiogenesis and cell survival. Am J Physiol Heart Circ Physiol. 2010; 299: H1772–81. 20870802 10.1152/ajpheart.00557.2010 PMC3006287

[43] Gruber R , Kandler B , Holzmann P , *et al* Bone marrow stromal cells can provide a local environment that favors migration and formation of tubular structures of endothelial cells. Tissue Eng. 2005; 11: 896–903. 15998229 10.1089/ten.2005.11.896

[44] Bronckaers A , Hilkens P , Martens W , *et al* Mesenchymal stem/stromal cells as a pharmacological and therapeutic approach to accelerate angiogenesis. Pharmacol Ther. 2014; 143: 181–96. 24594234 10.1016/j.pharmthera.2014.02.013

[45] Rahbarghazi R , Nassiri SM , Khazraiinia P , *et al* Juxtacrine and paracrine interactions of rat marrow‐derived mesenchymal stem cells, muscle‐derived satellite cells, and neonatal cardiomyocytes with endothelial cells in angiogenesis dynamics. Stem Cells Dev. 2013; 22: 855–65. 23072248 10.1089/scd.2012.0377 PMC3585743

[46] Costa PZ , Soares R . Neovascularization in diabetes and its complications. Unraveling the angiogenic paradox. Life Sci. 2013; 92: 1037–45. 23603139 10.1016/j.lfs.2013.04.001

[47] Page P , DeJong J , Bandstra A , *et al* Effect of serum and oxygen concentration on gene expression and secretion of paracrine factors by mesenchymal stem cells. Int J Cell Biol. 2014; 2014: 601063. 25614742 10.1155/2014/601063 PMC4295344

[48] Deschepper M , Oudina K , David B , *et al* Survival and function of mesenchymal stem cells (MSCs) depend on glucose to overcome exposure to long‐term, severe and continuous hypoxia. J Cell Mol Med. 2011; 15: 1505–14. 20716129 10.1111/j.1582-4934.2010.01138.x PMC3823195

[49] Dzhoyashvili NA , Efimenko AY , Kochegura TN , *et al* Disturbed angiogenic activity of adipose‐derived stromal cells obtained from patients with coronary artery disease and diabetes mellitus type 2. J Transl Med. 2014; 12: 337. 25491476 10.1186/s12967-014-0337-4 PMC4268805

